# How reliable is deep inspiration breath hold at CT simulation? Clinical impact of surface-guided breathing training in breast cancer radiotherapy

**DOI:** 10.1186/s13014-026-02920-y

**Published:** 2026-09-04

**Authors:** Lou Dietrich, Niklas Lackner, Andre Karius, Johann Brand, Rafael Lobao, Aliheydar Huseynli, Oliver J. Ott, Stefanie Corradini, Rainer Fietkau, Christoph Bert, Maya Shariff

**Affiliations:** 1 Department of Radiation Oncology, Universitätsklinikum Erlangen, Friedrich- Alexander-Universität Erlangen-Nürnberg (FAU), Erlangen, Germany; 2 Comprehensive Cancer Center Erlangen-EMN (CCC ER-EMN), Erlangen, Germany

**Keywords:** Surface guided radiation therapy, DIBH, CT simulation, Dose evaluation

## Abstract

**Purpose:**

Deep inspiration breath hold (DIBH) is widely used to reduce cardiac dose in left-sided breast radiotherapy. However, the reliability of DIBH at CT simulation, has not been systematically evaluated despite the importance of CT simulation as the basis for treatment planning. We investigated the clinical impact of surface-guided breathing training on DIBH reliability and cardiac dose sparing.

**Methods:**

In this retrospective cohort study, 115 breast cancer patients undergoing DIBH CT simulation were analyzed before and after implementation of surface-guided breathing training using an SGRT system. DIBH performance was quantified by partial lung volume increase (PLVI) and breath-hold variability. Correlations between external surface motion and internal DIBH depth were assessed. Cardiac dose metrics were compared, with a subgroup analysis for patients receiving lymphatic drainage area (LDA) irradiation.

**Results:**

Prior to training, insufficient DIBH CTs—including negative PLVI cases—were observed. After training implementation, all CT scans were acquired in intended breath-hold states, eliminating insufficient DIBH acquisitions. Mean PLVI increased by ~ 9%, and breath-hold variability decreased by ~ 26%. Sternum motion correlated most strongly with PLVI, with multiregion surface monitoring yielding the highest internal correlation. These improvements were reflected in significant reductions in cardiac dose, particularly in the LDA subgroup, with selected heart dose metrics reduced up to 96%, while maintaining target coverage.

**Conclusions:**

DIBH at CT simulation with surface-guided breathing training proves to be reliable. The training prevents planning CTs in wrong breathing states and thus enables clinically meaningful cardiac dose reduction in breast radiotherapy.

**Supplementary Information:**

The online version contains supplementary material available at https://doi.org/10.1186/s13014-026-02920-y.

## Introduction

Deep inspiration breath hold (DIBH) is a cornerstone technique for cardiac dose reduction in left-sided breast cancer radiotherapy [1, 2]. While its dosimetric benefit is well established, most studies addressing DIBH reliability have focused on the treatment course, while its behavior at computed tomography (CT) simulation—where all subsequent planning decisions originate—has received comparatively little attention [3–5]. Inadequate breath-hold performance at this early stage may compromise treatment quality throughout the entire radiotherapy course, yet is difficult to detect without objective monitoring [6].

However, it has also been found that the risk of heart diseases increases due to the exposure to radiation, especially for left-sided breast cancer [7]. This led to the development of the deep inspiration breath-hold (DIBH) technique, which has been introduced 25 years ago [8]. With this technique, the distance between breast and heart is maximized by inspirating during the irradiation, which results in a reduction of cardiac and lung doses [9–11]. Given this, DIBH has since become a standard procedure in left breast cancer therapy [12].

Managing this kind of breathing technique in a consistent and stable way has its challenges. In the past, systems like active breath control devices [8] or external markers [13] have been used to perform DIBH both at the computed tomography (CT) scanner as well as the treatment machine. Later, surface guidance systems (SGRT) were introduced [14], providing a solution without ionizing radiation for positioning and monitoring patients during treatment by employing optical light. Besides detecting involuntary movements of the patient, SGRT systems can also assist with breath control. For this reason, they have become an invaluable resource for guiding and monitoring the stability of DIBH patients [15, 16]. Such SGRT systems also have been introduced to computed tomography simulation workflows [17] and in 2022, VisionRT brought a new system for the CT scanner, SimRT, on the market.

After the evaluation of the SimRT SGRT-system at the CT scanner with phantoms in correlation to table movement and sag [18], the opportunity to introduce a SGRT-guided breathing training with SimRT opened up. Due to the dose sparing potential of the DIBH treatment for breast cancer [8–10], it is important to ensure that CT scans with an appropriate DIBH state are used for planning and further treatment. An SGRT-guided breathing training could lead to an improvement of the stability and depth of the DIBH state. These improvements could also result in dose reduction on relevant organs-at-risk (OARs).

For an optimal performed SGRT-guided breathing training, determining the best measurement procedure when using SimRT is also important. This includes the position of the surveillance region-of-interest (ROI) on the patient surface. Previously, the sternum was suggested for SGRT-ROI placement in DIBH patients because of its reproducibility [16]. However, patients vary in their respiratory manner, displaying either an abdominal-, thoracic- or mixed breathing motion, resulting in different observable surface movements along the body. The best correlating surface location for monitoring should therefore be determined.

Against this background, this study investigates the reliability of DIBH at CT simulation and evaluates the clinical impact of SGRT-guided breathing training. We evaluate the clinical impact of surface-guided breathing training using SimRT on breath-hold depth and stability, the relationship between external surface motion and internal lung expansion, and the resulting dose sparing of cardiac organs at risk. By focusing on CT simulation as a potential workflow bottleneck, this work aims to clarify the role of surface guidance in ensuring the full clinical benefit of DIBH in breast cancer radiotherapy.

## Methods

### Materials

The SOMATOM go.Open Pro scanner (Siemens Healthineers AG, Forchheim, Germany) was used for CT imaging. All DIBH CT scans used 3 mm slice thickness, with the exception of one scan with 5 mm slice thickness. All free breathing (FB) CT scans had a 5 mm slice thickness. CT reconstruction used a Br40 soft tissue kernel and S3 smoothing strength. The surface guidance system utilized at the imaging site is SimRT (VisionRT, London, United Kingdom), which reconstructs the 3D surface structure out of stereo images with the help of visible light [19]. The system provides a 5 × 5 cm^2^ area (in SimRT-terms: patch) in which the surveilled surface is tracked. It can be manually positioned on the previously recorded surface. Activation of the monitoring displays the anterior-posterior surface motion in the chosen patch, which can also be displayed to the patient by using the real-time coach (RTC) (VisionRT, London, United Kingdom). The RTC is a small monitor/mobile phone that can be fixed to the CT scanner table with an extension arm to visualize the surface motion amplitude to the patient in the form of a moving bar. This bar changes color when reaching the desired breathing state. The level of this target breathing state as well as a safety margin around it are defined by the medical staff in a preparation phase just prior to the scan. Trained radiotherapy technologists (RTT) guide the patient through the procedure, instructing them to achieve the optimal reproducible breath hold level.

In order to determine whether the breathing training with SimRT increases the volume between the heart and breast, the part of the lung between these two organs is analyzed. For that analysis, the deep learning segmentation model TotalSegmentator [20] (University Hospital Basel, Basel, Switzerland) was used to automatically contour individual lung lobes, as well as the heart and breast. For the clinical segmentation of the OARs which is used for treatment planning as well as the planning target volume (PTV), syngo.via RTImageSuite (Siemens Healthineers AG, Forchheim, Germany) was used. Of all the contoured structures, the heart, left anterior descending artery (LAD) and left ventricle are of interest for this study.

RayStation 12 A (RaySearch Laboratories, Stockholm, Sweden) was used for treatment planning. For data evaluation, extraction, statistics and visualization, Python 3.8 (Python Software Foundation, Wilmington, Delaware, United States), MATLAB R2019b and MATLAB 2024a (The MathWorks, Natick, United States of America) were used.

### Study structure

In this study, all female DIBH patients who were treated in our clinic in 2022 (61 patients) and 2023 (54 patients) were examined. The age of the patients was between 25 and 84 years old, with a weight between 49 and 110 kg.

The patients were separated into two cohorts. The first patient cohort includes all patients treated with DIBH in 2022 before the introduction of the abovementioned surface guidance system. In this cohort, no visually supported respiratory training was performed. After getting instructed by the RTT, these patients were guided auditory to hold their breath according to scheduled instructions from the CT scanner. Therefore, this cohort is referred to as the *no-training* cohort.

The second cohort included all patients treated in DIBH after the introduction of the surface guidance system at the start of 2023. This cohort includes the patients of 2023 and respiratory training was performed using the surface guidance system including visual feedback from the RTC at the CT scanner. RTTs were equipped with a standardized instruction manual for patient coaching to achieve an optimal DIBH state. The translated version of the instruction manual is provided (see Additional File 1). Breath-hold initiation and termination were guided by verbal auditory instructions provided by the RTTs. This cohort is hereafter called the *training* cohort.

For both cohorts, two CT scans were performed, one in DIBH and one in free breathing without breathing instructions. The DIBH scan is used for segmentation, dose calculation and for monitoring the breath hold during the treatment, while the FB scan is necessary for the initial positioning with SGRT at the linear accelerator (LINAC). In the case of negative results in the lung volume part of the analysis, the corresponding CT pairs were excluded from the following parts of the study because this indicates a CT acquisition in an unintended or insufficient breath-hold state. This was done for six patients in the no-training cohort in order to ensure the integrity of the study and draw the correct conclusions from the recorded data. Additionally, patients that received both scans, but were eventually not treated in DIBH were also excluded from the dose study. This is the case for two patients in the no-training cohort and one patient in the training cohort, resulting in 53 patients for both cohorts in the dose study.

The target volume shape varies considerably whether lymph drainage area (LDA) is included. Therefore, the cohorts were sorted according to LDA inclusion in the target volume and analyzed separately. For the no-training cohort, nine patients had the LDA in their target volume while 44 did not. For the training cohort, 21 patients had the LDA in their target volume and 32 did not.

All patient data used in this study were acquired as part of clinical routine and analyzed retrospectively. Retrospective data analysis is feasible without consent of an institutional research committee based on Art. 27(4) BayKrG (Bavarian Hospital Law). Patient consent was thus not obtained.

### Lung volumes

**Fig. 1:**
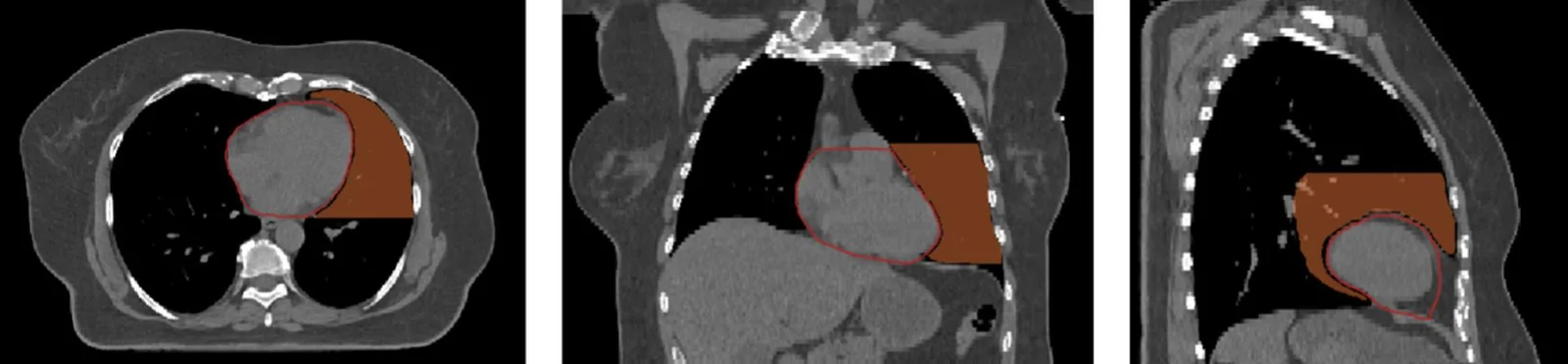
The partial lung volume (orange) used for analysis, shown for a patient’s deep inspiration breath hold scan. The determined partial lung volume is shown in the transversal, coronal and sagittal cut along the heart (red)

To analyze the effects of DIBH, the distance increase between heart and breast caused by inspiration was examined. For that, the lung volume between the left breast and heart was determined. The length and width as well as the most cranial slices of the heart contour were used to define the size of the partial lung volume (Fig. 1). The volume deviation between DIBH and FB scans was then calculated, which is referred to as partial lung volume increase (PLVI). Finally, the PLVI before and after the introduction of breathing training were compared to determine whether breathing training using SGRT with SimRT had an effect on DIBH depth, and thus, on the distance between the heart and the target volume [21].

In order to analyze the change in lung volume, the median partial lung volumes for both cohorts were calculated. The mean change in percent was calculated as well as the standard error, excluding faulty data points to ensure integrity. The stability of the DIBH state between the two cohorts was evaluated by calculating the median change between the DIBH median absolute deviations. In order to assess whether the changes due to the introduction of SGRT-based breathing training with SimRT were statistically significant, a two-sample t-test at a 5% significance level was performed.

### Respiratory manner and surface motion

Observing the surface motion allows conclusions to be drawn about the respiratory manner of the patient and, with that, the respiratory phase. An abdominal respiratory manner results in surface motion in the abdomen area [22], while a thoracic respiratory manner results in more surface motion in the upper chest-region [23]. In order to analyze respiratory manners and the influence of the breathing training with SimRT on them, the surface motion in these regions was evaluated from the CT scans for all patients. Additionally, the surface motion on the sternum region was analyzed, to cover a mixture of both respiratory manners.

**Fig. 2:**
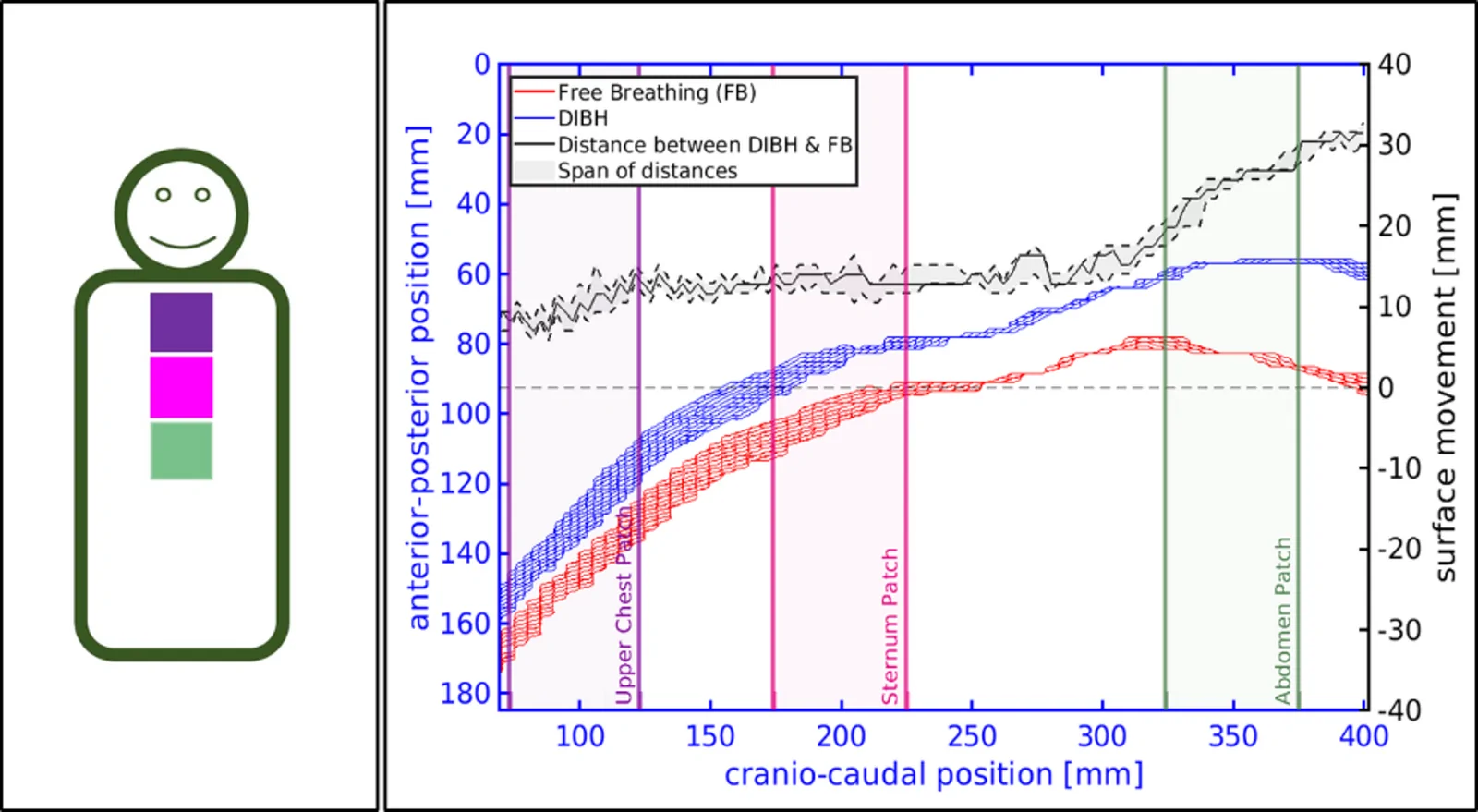
Placement of surveilled patches on an example patient (left), equal in size to the SimRT patch (5 × 5 cm^2^). The surface position of the patient was extracted from the CT scans starting from the clavicles, both for the free breathing (FB) (red) and deep inspiration breath hold (DIBH) (blue) scan (right). The surface movement was surveilled in the upper chest (purple), sternum (pink) and abdomen (green) patches. The distance was calculated separately for each sagittal and longitudinal slice, with the median of them resulting in the black line. The gray area portrays the minima and maxima of surface movement for each longitudinal slice

The movement of the patient surface on the upper chest, sternum and abdomen was calculated. For that, analyzed surface areas were defined with the same width and length as the SimRT patch and were positioned along the central sagittal slices of the CT scan for each patient. The placement of these patches along the patient surface can be seen for an example case in Fig. 2. Since the slice distance in sagittal direction is 1 mm, 50 sagittal slices were used to calculate the median surface position of each longitudinal CT slice. In Fig. 2, each of the 50 FB sagittal slices is represented by a red line, and each of the 50 DIBH sagittal slices by a blue line.

To determine the surface movement (black line in Fig. 2), the difference of the median surface position between DIBH and FB CT was calculated for each longitudinal slice. To determine the movement for the three investigated surface areas, the median surface movement of all longitudinal slices in each patch was calculated. Difficulties in producing the identical patch position between the DIBH and FB CT scan of each patient could be avoided due to both CT scans of each patient being identical in size.

Calculating the difference between the FB and DIBH state represents the DIBH amplitude gained for each region when performing DIBH. Since, according to Guy et al. [24], the sum of the recorded surface motion correlates with the tidal lung volume — the air volume entering and exiting the lung during a respiratory cycle — the sum of the surface motion of the three patches was also analyzed. This was done in order to see if the observation of multiple patches would be more beneficial compared to only one patch.

In order to analyze if the breathing training with SimRT resulted in an increase of the surface motion, two-sample t-tests at a significance level of 5% were performed. The correlation between PLVI and the surface motion in the area of the three patches was investigated using the Pearson’s correlation coefficient with a significance level of 5%, according to Cohen et al. [25].

### Dose evaluation

The planning target volume (PTV) and OARs (heart, left anterior descending artery and left ventricle) were contoured. The segmentation for the OARs was performed by DirectORGANS, a feature of the CT scanner to avoid implementing a human bias. The contours were manually evaluated on correctness afterwards. The PTV was contoured as described in Ott et al. [26]. Structure of OARs that could not be automatically delineated, for example the LAD, were contoured manually by an assistant physician and checked by an attending physician under the four-eye principle.

After that, the dose distribution was calculated following the in-house clinic standard. The prescribed dose regimen was either 28 fractions of 180 cGy or a hypofractionated regimen of 15 fractions of 267 cGy. The dose goals for the OARs follow the RTOG (Radiation Therapy Oncology Group)/EORTC (European Organisation for Research and Treatment of Cancer) guidelines and the suggested clinical goals by Duma et al. [27]. The dose distribution was checked and approved by an attending physician. The treatment plans were either 3DCRT (Three-Dimensional Conformal Radiation Therapy), IMRT (Intensity-Modulated Radiation Therapy), VMAT (Volumetric Modulated Arc Therapy) or a combination of them. The decision on which modality was used was decided case by case by the planning medical physicist in order to achieve the above-mentioned clinical goals, with a preference for 3DCRT and IMRT.

To analyze the improvement of the dose in the cardiac structures, the mean dose of the heart (H mean), mean LAD (LAD mean) and mean left ventricle (LV mean) dose, as well as the volumes of the left ventricle that receive 5 Gy and 23 Gy ($\:{LV}_{V5}$, $\:{LV}_{V23}$), were extracted and compared between the two cohorts. These dose limits are taken from Duma et al. [27]. Since there were different fractionation schemes, the doses were transferred into 2 Gy per fraction equivalent dose ($\:{\mathrm{EQD}}_{\mathrm{2}}$) using alpha/beta = 3 Gy according to the linear quadratic model.

The planning target volume coverage was also analyzed to deduce whether a potential reduction of dose to the organ structures leads to a decline in coverage. In order to analyze whether the introduction of a surface guided breathing training with SimRT had an impact on the dose, two-sample t-tests at a 5% significance level (for normal distributed data or cohorts bigger than 30 data points) or Mann-Whitney U tests at a 5% significance level (for non-normally distributed data or cohorts smaller than 30) were conducted.

## Results

### Lung volumes

**Fig. 3:**
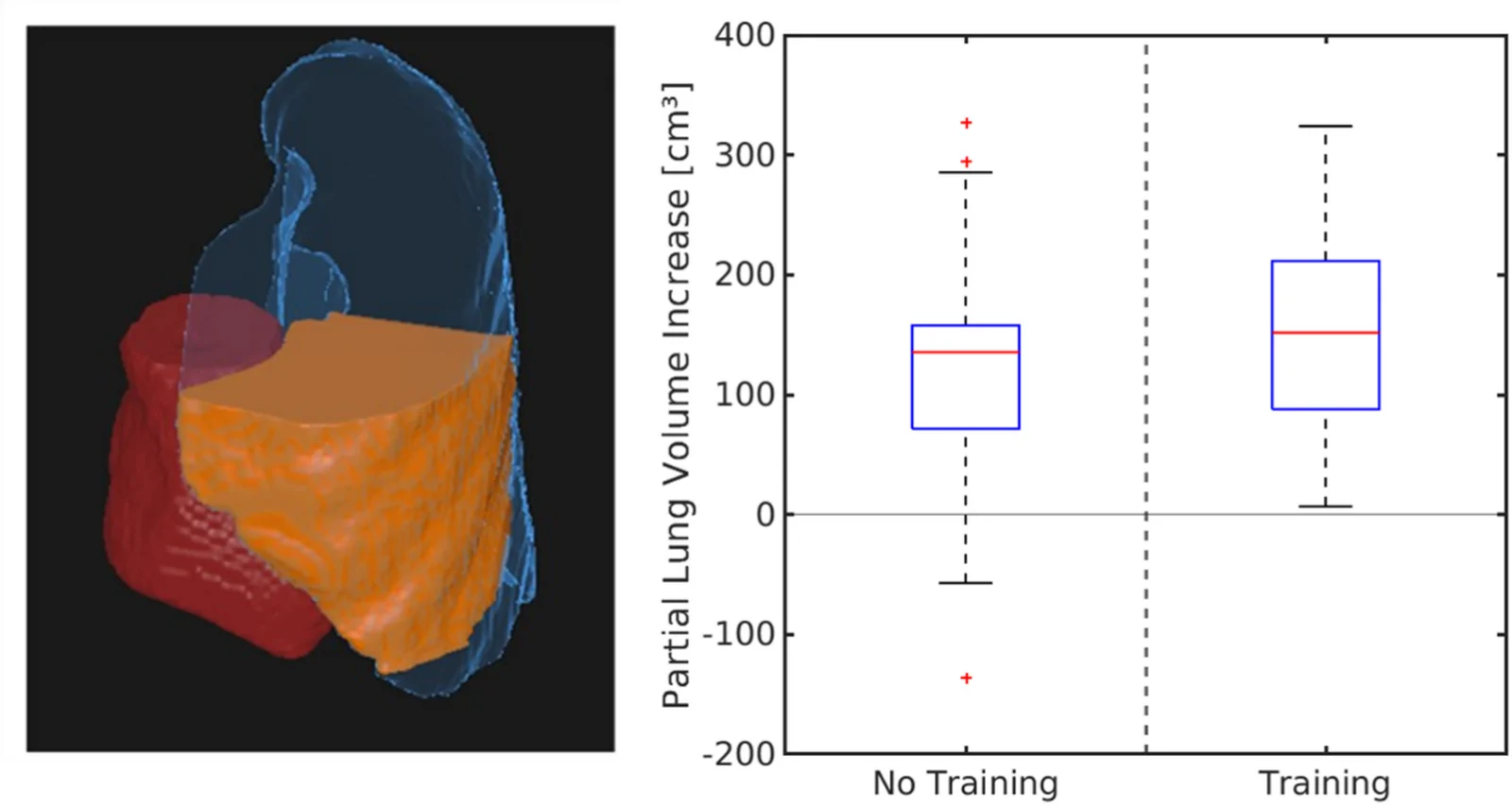
The volume of the left lung (light blue) along the length of the heart (dark red) was calculated. The resulting volume is called the partial lung volume (orange). The difference of this volume between deep inspiration breath hold and free breathing was determined, resulting in the partial lung volume increase (PLVI). This partial lung volume increase is shown for the training and no-training cohorts (right)

To investigate if the lung volume increased, the PLVI of the part of the lung that is located between the heart and the left breast was determined. The median partial lung volume measured for the DIBH state and the FB state as well as the PLVI are shown in Table 1. The PLVI before and after the training are shown in Fig. 3.

An improvement in partial lung volume can be seen for the training cohort in comparison to the no-training cohort by ($\:9\pm\:1$) % ($\:{\mathrm{p}}_{\mathrm{PLVI}}$ = 0.04). The median absolute deviation of the partial lung volume in DIBH was also reduced by 26% compared to the no-training cohort.

**Table 1:** Median lung volume and median absolute deviation in deep inspiration breath hold and free breathing along the region of the heart, as well as the increase of the lung volume (PLVI) between the two breathing states

Cohort	DIBH [$\:{\mathrm{cm}}^{3}$]	FB [$\:{\mathrm{cm}}^{3}$]
no-training	$\:(639.0\pm\:119.3)$	$\:(525.1\pm\:107.4)$
training	$\:(666.1\pm\:94.8)$	$\:(518.8\pm\:102.6)$

### Respiratory manner and surface motion

To assess whether breathing training with SimRT at the CT scanner had an impact on the surface motion of the patient, the median and median absolute deviation of the patches on the upper chest, sternum and abdomen were compared between the two cohorts. Additionally, the sum of the surface motions of all three patches was analyzed. The results of these measurements are summarized in Table 2. Movement in the upper chest patch was the lowest considering both cohorts ((2.3 $\:\pm\:$ 3.8) mm and (4.7 $\:\pm\:$ 5.5) mm) while the abdomen patch showed the largest motion amplitude ((15.2 $\:\pm\:$ 6.8) mm and (16.4 $\:\pm\:$ 7.1) mm).

**Table 2:** Median and median absolute deviation of the surface movement recorded in the different body regions and the sum of all three regions for the training and no-training cohorts. These values show how much the surface in the named region moved in median between the DIBH and FB state

Cohorts	Upper Chest [mm]	Sternum [mm]	Abdomen [mm]	Sum [mm]
no-training	(2.3$\:\pm\:$3.8)	(5.9$\:\pm\:$3.5)	(15.2$\:\pm\:$6.8)	(23.1$\:\pm\:$9.5)
training	(4.7$\:\pm\:$5.5)	(8.8$\:\pm\:$4.6)	(16.4$\:\pm\:$7.1)	(29.3$\:\pm\:\:$12.4)

Following that, the motion in the three selected areas on the surface of the patient was correlated with the PLVI for each individual patient using the Pearson’s correlation coefficient. The results are shown in Table 3. The values indicate that the strongest correlation between the PLVI and the surface motion was found for the sternum region. According to Guy et al. [24], the tidal lung volume correlates with the sum of all acquired surface motions, therefore it was tested if this statement also holds for the patients selected in this study and considering the calculated partial lung volume. This analysis showed that in both cohorts there has been a moderate correlation between the sum of the three surface patches and the PLVI ($\:{\mathrm{r}}_{\mathrm{SAP(22)}}$=0.35 ($\:\mathrm{p}$=0.009) in 2022 and $\:{\mathrm{r}}_{\mathrm{SAP(23)}}$=0.47 ($\:\mathrm{p<}$0.001) in 2023).

**Table 3:** Pearson’s correlation coefficient between surface patch movement, the sum of all surface patches and the increase of the partial lung (PLVI). It shows moderate and low correlation for all three patches as well as moderate correlation for the sum of all patches over the various cohorts. *p* < 0.05 for all shown correlations

Cohort	Upper Chest$\:{\mathrm{r}}_{\mathrm{UC}}$	Sternum$\:{\mathrm{r}}_{\mathrm{ST}}$	Abdomen$\:{\mathrm{r}}_{\mathrm{AB}}$	Sum$\:{\mathrm{r}}_{\mathrm{SAP}}$
no-training	0.34 ($\:\mathrm{p}$=0.01)	0.43 ($\:\mathrm{p}$=0.001)	0.31 ($\:\mathrm{p}$=0.02)	0.35 ($\:\mathrm{p}$=0.009)
training	0.33 ($\:\mathrm{p}$=0.01)	0.41 ($\:\mathrm{p}$=0.002)	0.29 ($\:\mathrm{p}$=0.03)	0.47 ($\:\mathrm{p}$<0.001)

### Dose study

For the dose study, the effect of the training on the EQD2 doses and volumes receiving dose of the heart, as well as the cardiac structures LAD and left ventricle, were analyzed. On top of that, the change on the target coverage was investigated.

**Fig. 4:**
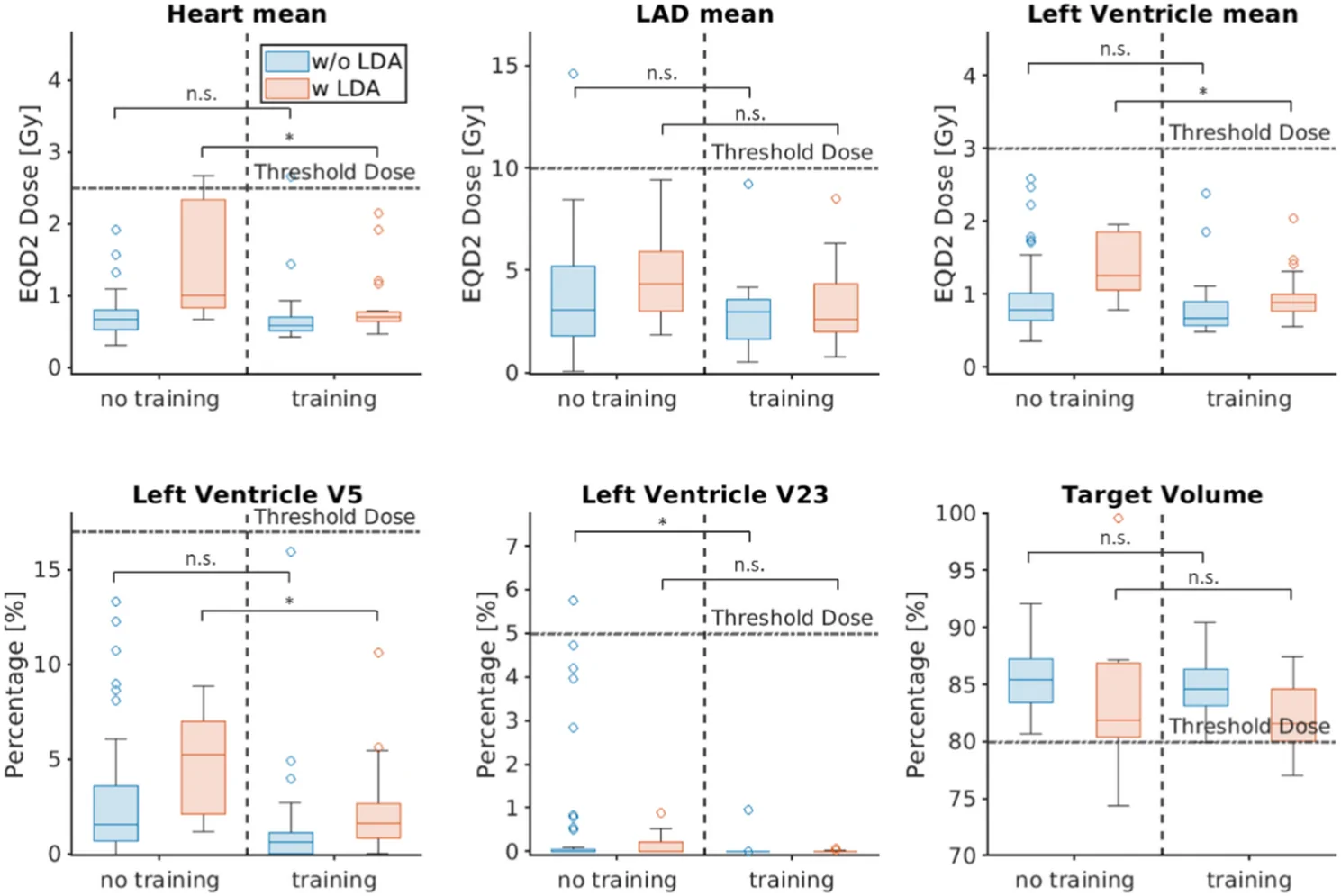
EQD2 mean dose for the heart, LAD, and left ventricle, as well as the left ventricle V5 and V23 and planning target volume coverage for the no-training and training cohorts. The data was also separated depending on the LDA inclusion in their target volume. The dashed-dotted gray line shows the clinical threshold dose, which was the maximum dose the OAR should receive or the volume that should receive a certain dose threshold, taken from Duma et al. [27]. For the planning target volume it was the minimum required coverage. There was a significant improvement (*) for several OARs, mainly for the patients with LDA-inclusion. The coverage of the planning target volume did not change significantly for both patient groups

The patients which had the LDA in their target volume, were analyzed separately from those without LDA inclusion. The mean doses and volumes at a certain dose received by the risk structures are displayed with the in-house clinically used threshold dose in Fig. 4. When analyzing dose, the threshold marks the maximum accepted dose for an in-house clinical goal. When analyzing volumes, the threshold volume percentage marks the maximum accepted volume that receives a certain dose for an in-house clinical goal. Values above that threshold dose or volumes are only accepted in exceptions.

There was a significant reduction of the EQD2 mean dose and volume for OARs due to the breathing training introduction with SimRT in 2023, mainly for the patient group with LDA in the target volume. For the patients with LDA inclusion, the mean of the heart mean dose was reduced by ($\:40\pm\:13)$ % (*p* = 0.01), the left ventricle mean dose was reduced by ($\:29\pm\:9$) % (*p* = 0.02) and the volume of $\:{LV}_{V5}$ was reduced by ($\:54\pm\:14$) % (*p* = 0.02). Although not statistically significant, the mean of the EQD2 LAD mean dose was reduced by ($\:31\pm\:14$) % (*p* = 0.08) and the volume of $\:{LV}_{V23}$ by ($\:96\pm\:3$) % (*p* = 0.08) but on a level with very low absolute values. For the patients without the LDA in their target volume, there was a significant reduction of the volume of $\:{LV}_{V23}$ by ($\:95\pm\:6$) % (*p* = 0.04) due to the introduction of the training. Even though the changes were not statistically significant, the EQD2 heart mean dose was reduced by ($\:6\pm\:12$) % (*p* = 0.62), the EQD2 LAD mean dose by ($\:23\pm\:11$) % (*p* = 0.11), the EQD2 left ventricle mean dose by ($\:17\pm\:10$) % (*p* = 0.14) and the volume of $\:{LV}_{V5}$ by ($\:50\pm\:20$) % (*p* = 0.05) after the introduction of the training.

Regarding planning target volume coverage, training did not significantly affect the outcome. For both groups, the coverage did not change significantly with the coverage for the patients without LDA inclusion, having a mean coverage of $\:(86\pm\:3)$ % before the breathing training introduction and a mean coverage of $\:(85\pm\:3)$ % after the introduction (*p* = 0.36). For the patients with LDA inclusion in the target volume, the mean coverage was $\:(84\pm\:7)$ % before the breathing training introduction and $\:(82\pm\:3)$ % coverage after the breathing training introduction (*p* = 0.27).

## Discussion

The aim of this study was to investigate the influence of SGRT-guided breathing training with SimRT on DIBH performance at CT simulation compared to auditory instructions alone. The results demonstrate that SGRT significantly improves DIBH reliability by increasing breath-hold stability and preventing incorrectly acquired CT scans, addressing a critical step in the radiotherapy workflow that directly impacts downstream treatment quality. In the no-training cohort, six cases with negative PLVI indicated that CT scans were not acquired in a proper DIBH state. This suggests that CT acquisition may have been initiated before patients reached a stable breath-hold level, potentially due to delayed inspiration following automated auditory instructions. After implementation of SGRT, RTTs were able to monitor the breathing level in real time and manually trigger CT acquisition once a stable DIBH state was achieved. Consequently, no insufficient DIBH CT acquisitions were observed in the following year, indicating effective elimination of this error source. These findings highlight CT simulation as a critical but previously underrecognized source of variability in DIBH workflows, despite its central role in treatment planning. The observed increase in PLVI after SGRT implementation was accompanied by increased surface motion, particularly in the thoracic region, consistent with known respiratory mechanics. The correlation analysis indicates that multi-region surface monitoring can serve as a surrogate for internal lung expansion, in agreement with previous work on surface-guided DIBH monitoring. In line with Everitt et al. [28], the correlation between PLVI and abdomen motion remained stable between cohorts, suggesting no influence of breathing training in this region. For the other patches and the combined surface signal, coefficients of variance below 20% do not allow exclusion of a training-related effect. However, the sample size remained below the recommended threshold for correlation analysis as described by Bonett et al. [29]. The moderate correlation observed between surface motion and heart–breast distance is consistent with findings by Wu et al. [30]. Overall, SGRT-guided training increases DIBH amplitude without fundamentally altering the relationship between external motion and internal lung expansion.

The dosimetric analysis demonstrates that these improvements translate into clinically relevant dose reductions, particularly for patients with LDA inclusion, aligning with the established goal of DIBH to reduce cardiac exposure [31]. While no significant reduction was observed for the LAD, this may be explained by its small volume relative to other OARs, making it more sensitive to localized dose variations. The increased motion amplitude in the upper thorax following SGRT implementation provides a plausible explanation for the observed dose reduction in patients with LDA involvement, as this region is directly affected by improved DIBH depth. For patients without LDA inclusion, dose reductions were less pronounced but consistently observed across all evaluated parameters. Importantly, these improvements were not associated with a reduction in PTV coverage, confirming that the observed dosimetric benefits are not achieved at the expense of target coverage. In addition to eliminating insufficient CT acquisitions, SGRT-guided breathing training therefore enhances the achievable dose distribution in DIBH treatments and may contribute to reduced treatment-related toxicity [31].

This study focuses on the impact of SGRT-guided breathing training at CT simulation. Other workflow modifications, such as introducing a delay between training and CT acquisition, have been shown to have no significant impact on cardiac or lung dose [32]. However, the full clinical benefit of SGRT-based DIBH workflows also depends on treatment delivery at the LINAC. Accurate patient positioning and stable breath-hold reproducibility during treatment are essential for consistent dose delivery [33, 34]. Future studies should therefore investigate whether SGRT-guided training at CT simulation improves DIBH reproducibility and treatment quality at the LINAC.

Another point that should be mentioned is that while this study highlights advantages of DIBH monitoring with SGRT, the acquisition costs of SGRT systems are relatively high. Alternatives such as spirometry-based systems, optical marker tracking, or video-based monitoring [35, 36] are available that were not analyzed as part of this study but provide adequate DIBH monitoring at likely lower costs. When thinking about implementing this type of system, the full spectrum of clinical applications of each available system needs to be considered. Beyond DIBH monitoring alone, capabilities like patient positioning, intrafraction motion monitoring, and applicability across multiple treatment sites should be factored into the acquisition assessment.

## Conclusion

This study demonstrates that DIBH at CT simulation may be susceptible to unintended breath-hold states when relying on auditory instructions alone. The implementation of SGRT-guided breathing training effectively eliminated insufficient DIBH CT acquisitions and improved breath-hold depth and stability. These improvements translated into clinically relevant reductions in cardiac dose, particularly for patients with LDA inclusion, without compromising target coverage.

By addressing a critical source of variability at CT simulation, SGRT-guided training enhances the robustness of the entire DIBH workflow and supports more consistent delivery of cardiac-sparing radiotherapy.

## Supplementary Information

Below is the link to the electronic supplementary material.

Supplementary Material 1: Additional File 1: File name: ReliabilityofDIBHatCTsim_additional_file_1.docx. Title of data: INSTRUCTIONS FOR DIBH AT CT SCANNER – SimRT. Description: The additional file provides a translated version of the instruction manual given to the staff on how to use the SimRT and how to perform the DIBH training and CT simulation when using SimRT

## Data Availability

The datasets used and/or analyzed during the current study are available from the corresponding author on reasonable request.
