# Supplementary material for: How reliable is deep inspiration breath hold at CT simulation? Clinical impact of surface-guided breathing training in breast cancer radiotherapy
Source: Radiat Oncol. 2026 Sep 4;21:120. doi: 10.1186/s13014-026-02920-y (PMC13543520; doi:10.1186/s13014-026-02920-y)
Supplement: Supplementary file 1 [file 13014_2026_2920_MOESM1_ESM.docx]

INSTRUCTIONS FOR DIBH AT CT SCANNER – SimRT

Calibration & Daily QA:

- Daily QA:
- Daily before the first patient
- Start the software and select “Daily QA”
- Place the calibration board as close as possible to the bore with the arrow on the calibration board pointing in the direction of the bore on the CT table
- Pre-position the board on the outbore laser (x, y: centre, z: upper edge of the board)
- Move the board in bore, position with the inbore laser
- QA check a): translation check, start the software “QA check”:
  🡪 if successful: continue
  🡪 if unsuccessful: start again from the beginning
  🡪 if the error occurs again: calibration necessary.
- Move CT table 250 mm into bore
- QA check b): rotation check, tap ‘Continue’ at the top
  🡪 if successful, remove QA check, finish
  🡪 if unsuccessful: calibration necessary
- Calibration:
- Every 3 weeks
- works the same as the daily QA check

Patient training instructions:

- Step 1: Start treatment: Patients 🡪 select ‘Patient’ or create patient case for new patient 🡪 select DIBH protocol
- Step 2: Explain procedure to patient and practice DIBH several times without any visual feedback
- patient should inhale as deeply as possible on command while also being able to hold the breath at least 20 seconds uninterrupted
- Step 3: Set up patient with inbore laser 🡪 press ‘Capture’ in SimRT software
- Step 4: Position monitoring patch 🡪 position on the sternum if possible 🡪start ‘Tracking’ 🡪 Real Time Coach (RTC) works now and shows to the patient:
- A white line: This symbolises the target DIBH range
- A thick green/yellow bar, yellow when outside the target DIBH range, green when in the DIBH range
- Step 5: practice several times with the RTC on 🡪 adjust patch position if necessary, then set the DIBH range 🡪 range size can be adapted if necessary 🡪 remember position of the range
- Step 6: explain RTC to patient and practice again with giving breathing commands 🡪 repeat several times and adjust inspiration range if necessary 🡪 consider recovery time to return to normal resting breathing after DIBH
- Step 7: topogram: surface tracking must be switched off because table speed is faster🡪 the patient must follow the auditory commands of RTTs, avoid automated commands by the scanner
- Step 8: return to original position 🡪 perform ‘capture’ and activate monitoring 🡪 set DI range to previous one 🡪 reevaluate DI range position by letting patient perform DIBH in order to validate
- Step 9: Run the CT scan 🡪 the breathing curve might move during table movement, this is caused by table bending 🡪 turn off the tracking after the DIBH CT scan 🡪 give command to continue breathing
- Additional note: if contrast agent is involved, reevaluation for DI range position becomes not possible
